# COVID-19 and Pulmonary Emboli: A Case Series and Literature Review

**DOI:** 10.5811/cpcem.2020.7.48174

**Published:** 2020-07-16

**Authors:** James Greenan-Barrett, Adrian Perera

**Affiliations:** Royal Free Hospital, Department of Emergency Medicine, Hampstead, London, United Kingdom

**Keywords:** Pulmonary embolus, COVID-19

## Abstract

**Introduction:**

There is recent evidence that coronavirus disease 2019 (COVID-19) infection results in a prothrombotic state that may increase the risk of venous thromboembolism. Both COVID-19 infection and pulmonary emboli can present with dyspnoea, tachypnoea, hypoxaemia and an elevated D-dimer. Identifying a pulmonary embolus in a patient with COVID-19 and differentiating it from the typical clinical and biochemical features of COVID-19 is challenging.

**Case Reports:**

We report four cases, and reviewed two further cases in the literature, of a pulmonary embolus in patients who presented to the emergency department with COVID-19 and no other risk factor for a pulmonary embolus.

**Conclusion:**

We identified a series of atypical features that should raise suspicion for a pulmonary embolus: pleuritic chest pain; haemoptysis; atrial fibrillation; tachycardia; hypotension; late onset deterioration; evidence of right heart strain; or a disproportionally elevated D-dimer in comparison to ferritin.

## INTRODUCTION

Coronavirus disease 2019 (COVID-19) is a novel disease that usually presents with mild symptoms; however, in 14% of patients it can result in a severe disease requiring hospitalisation.^1^ The severe form of the disease is characterised by severe hypoxaemia that is predominantly thought to be secondary to acute respiratory distress syndrome (ARDS).^2^ There have been a limited number of reports of pulmonary emboli in COVID-19 patients which may also contribute to hypoxaemia.^3^–^6^ However, the significant crossover between the presenting features of COVID-19 and pulmonary emboli makes differentiating this cohort challenging. We report four cases of COVID-19 complicated by a pulmonary embolus, and we analysed the literature to establish common “red flag” features that should raise clinical suspicion for a pulmonary embolus.

## CASE SERIES

### Case 1

A 72-year-old woman presented to the emergency department (ED) after she attended the hospital for a routine ophthalmology appointment and felt dyspnoeic. She had experienced one week of lethargy, feeling generally unwell and loss of taste, and one day of dyspnoea, palpitations, and diarrhoea. She did not complain of fevers, cough, or chest pain. She was entirely independent and had a history of hypertension, seasonal asthma, and glaucoma. On assessment she was tachypnoeic (32 breaths per minute) with increased work of breathing and had oxygen saturations of 90% on 15 litres of oxygen via a non-rebreather mask. She was in atrial fibrillation with a rapid ventricular response (125–170 beats per minute) and was hypotensive (87/62 millimetres of mercury [mmHg]).

Venous and arterial blood samples were taken and sent for analysis (Table 1). A chest radiograph (CXR) showed widespread bilateral infiltrates suspicious for COVID-19. A computed tomography pulmonary angiogram (CTPA) demonstrated extensive bilateral pulmonary emboli with no evidence of right heart strain and extensive, patchy ground-glass changes in keeping with COVID-19. She was given intravenous (IV) fluids, magnesium sulphate and verapamil which reduced her heart rate to 115 beats per minute, but she remained in atrial fibrillation and remained hypotensive (95/60 mmHg). She was chemically cardioverted with IV amiodarone although she remained hypotensive. She was given treatment dose tinzaparin before being switched to rivaroxaban. Her case was discussed with interventional radiology, cardiology, and intensive care regarding catheter-directed and systemic thrombolysis; a decision was made that thrombolysis was not appropriate due to the risk of pulmonary haemorrhage and that the persistent hypotension was likely related to verapamil therapy. A nasopharyngeal aspirate confirmed COVID-19 infection on reverse transcription-polymerase chain reaction (RT-PCR).

### Case 2

A 62-year-old man was brought into the ED by ambulance with three days of worsening dyspnoea, reduced appetite, myalgia, intermittent diarrhoea, abdominal cramping and one episode of vomiting, but no chest pain. He had experienced a fever three weeks previously and tested positive for COVID-19 before self-isolating and completing a course of azithromycin. He was previously independent and his only medical history was hypertension and hypercholesterolaemia. On assessment in the ambulance he had oxygen saturations of 52% on room air which improved to 88% on 15 litres of oxygen via a non-rebreather mask. In the ED he was tachypnoeic (24 breaths per minute), with saturations of 97% on 15 litres of oxygen. He was tachycardic (113 breaths per minute), normotensive (100/72 mmHg), and an electrocardiogram (ECG) showed sinus tachycardia with no evidence of right heart strain.

A CXR showed extensive, bilateral peripheral patchy opacification suspicious for COVID-19. A CTPA demonstrated bilateral acute pulmonary emboli with no features of right heart strain and extensive peripheral ground-glass and peri-lobular consolidation consistent with COVID-19. The patient was treated with treatment dose tinzaparin and high-flow oxygen. A nasopharyngeal aspirate confirmed COVID-19 infection RT-PCR.

### Case 3

A 78-year-old man was brought into the ED by ambulance with four days of fever, dry cough, lethargy, myalgia, coryza, dyspnoea and one episode of haemoptysis, but no chest pain. He had a history of benign prostate hyperplasia and hypercholesterolaemia, but was otherwise active and independent. On assessment with the ambulance service he was in respiratory distress and had oxygen saturations of 60% on room air. On assessment in the ED he had oxygen saturations of 88% on 15 litres of oxygen via a non-rebreather mask and tachypnoeic (35 breaths per minute). He was normotensive (120/94 mmHg) but had cool peripheries with a peripheral capillary refill time of five seconds. An ECG showed he was in atrial fibrillation with a rapid ventricular response (140 beats per minute) with ST depression in lateral leads and no evidence of right heart strain.

**CPC-EM Capsule**

What do we already know about this clinical entity?

Coronavirus disease 2019 (COVID-19) infection can cause significant hypoxaemia that is predominantly thought to be secondary to acute respiratory distress syndrome.

What makes this presentation of disease reportable?

These patients with COVID-19 infection developed pulmonary emboli; these cases highlight atypical features that should raise suspicion for a pulmonary embolus.

What is the major learning point?

Emergency clinicians should consider a pulmonary embolus in patients with COVID-19 who present with atypical clinical and biochemical features.

How might this improve emergency medicine practice?

This may reduce the rate of undiagnosed pulmonary emboli that can be treated to improve outcomes in patients with COVID-19.

A CXR showed bilateral consolidation suspicious for COVID-19. A point-of-care echocardiogram showed reduced right ventricular free wall contractility. A CTPA showed multiple subsegmental pulmonary emboli throughout the right hemithorax and left lower lobe with evidence of right heart strain (right ventricle:left ventricle ratio >1:1) and extensive ground-glass changes. The patient was taken to the intensive care unit (ICU) due to progressive hypoxia where he was intubated and ventilated and treated with treatment dose tinzaparin. A nasopharyngeal aspirate confirmed COVID-19 infection RT-PCR.

### Case 4

A 63-year-old man presented with two weeks of myalgia, fever and lethargy, and three days of haemoptysis and pleuritic chest pain. He was independent with no comorbidities. He had normal observations and a normal examination. An ECG showed new right bundle branch block. A CXR showed bilateral peripheral areas of consolidation suspicious for COVID-19. A CTPA demonstrated bilateral pulmonary emboli with complete occlusion to the left lower lobar artery, a right sided pulmonary infarct, right heart strain (right ventricle:left ventricle ratio of 1.4:1), and patchy ground-glass shadowing suggestive of COVID-19. He was treated with treatment dose tinzaparin.

## DISCUSSION

There has been one case report of a pulmonary embolus in a patient with COVID-19 who was identified after an echocardiogram demonstrated right heart strain (dilated + hypokinetic right ventricle with a raised pulmonary arterial pressure).^3^ Another case report documents a patient with chest pain, haemoptysis, and ECG evidence of right heart strain (S1Q3T3 pattern and right-axis deviation).^4^ Table 2 compares the clinical and biochemical features of the four patients that we report and the two further cases in the literature.

In the context of COVID-19, raising clinical suspicion for a pulmonary embolus and distinguishing it from the typical features of COVID-19 is difficult as 30% of inpatients with COVID-19 are dyspnoeic, 76% are hypoxaemic, and 29% are tachypnoeic (respiratory rate >24 breaths per minute).^2^,^7^ However, only 1–2% of inpatients with COVID-19 present with hypotension (systolic blood pressure <90 mmHg), tachycardia (heart rate >125 beats per minute), chest pain, or haemoptysis.^8^ Therefore, it is important to consider a pulmonary embolus in patients with COVID-19 who present with these atypical features. Additionally, there are no published cases of patients presenting with new-onset atrial fibrillation. Features of right heart strain on ECG and echocardiogram are also seen in pulmonary emboli, due to a rapid increase in pulmonary vascular resistance, and a pulmonary embolus should be excluded. The median time from illness onset to dyspnoea was eight days (interquartile range 5–13) and to admission to the ICU was 12 days (interquartile range 8–15). Any patients with late onset dyspnoea should be assessed further for a pulmonary embolus.^7^

All four patients we reported had significantly elevated D-dimer values; however, as both a marker of thrombosis and acute inflammation, the diagnostic value of the D-dimer is poor. Ninety percent of inpatients with COVID-19 have an elevated D-dimer, although a value of >1000 nanograms per microlitre (ng/mL) (normal range <400 ng/mL) is an independent risk factor for death.^7^ In a study of 25 patients with COVID-19 who received a CTPA, the 10 patients who were diagnosed with acute pulmonary emboli had on average significantly higher D-dimer values than those who did not (11,070 v 2440 ng/mL).^4^ In another study, 25 out of 81 COVID-19 patients on the ICU had a lower limb deep vein thrombosis, and the strongest correlator was D-dimer; a value of >1500 ng/mL predicted a deep vein thrombosis with 85% sensitivity and 89% specificity.^9^ The patients we reported had significantly elevated D-dimer values of between 9892–>80,000 ng/mL (normal range <400 ng/ml), equating to a 25–200 fold increase above the upper limit of normal.

Ferritin is another acute phase protein that is elevated in patients with COVID-19, with higher levels in non-survivors than with survivors but does not appear to be elevated as a result of venous thrombosis.^7^ The patients we reported had modestly elevated ferritin values of between 871–2197 micrograms per litre (ug/L) (normal range 10–350 ug/L), equating to a 2–6 fold increase above the upper limit of normal. Thus, we suggest that in a patient with COVID-19, a disproportionally elevated D-dimer in comparison to ferritin should raise suspicion for a pulmonary embolus.

Concerns have been raised of using contrast in a patient population that may be at increased risk of acute kidney injury.^3^ However, in a study of 116 COVID-19 confirmed patients, none of them met the criteria for diagnosis of an acute kidney injury and none of the patients we reported had a deterioration in renal function after contrast.^10^ Thus, we suggest that in a patient with sufficient renal function, that a CTPA should not be avoided if a pulmonary embolus is suspected.

In all cases in this series, there were no pre-existing risk factors for venous thromboembolism, although COVID-19 itself may be a risk factor. Seventy-one percent of non-survivors with COVID-19 met the criteria for disseminated intravascular coagulation which appears to be predominantly prothrombotic.^11^–^12^ Post-mortem lung dissection found microvascular thrombosis in a COVID-19 patient and a series of patients with the related SARS-CoV1.^13^–^14^ A case series of three COVID-19 patients found significant coagulopathy with peripheral ischaemia and bilateral cerebral infarctions in multiple vascular territories; notably all three patients had positive antiphospholipid antibodies.^15^

All patients were treated with treatment dose tinzaparin whilst thrombolysis was avoided due to the risk of haemorrhage. One study found that heparin prophylaxis in COVID-19 positive inpatients with a D-dimer >3000 ng/L reduced the 28-day mortality.^11^

## CONCULSION

We reported four cases of COVID-19 patients presenting with an acute pulmonary embolus. It is important to be aware of atypical features of COVID-19 infection that may be more suggestive of a pulmonary embolus such as pleuritic chest pain, haemoptysis, atrial fibrillation, tachycardia, hypotension, late onset deterioration, evidence of right heart strain, or a disproportionally elevated D-dimer in comparison to ferritin.

Further investigation is required into the role of prophylactic anticoagulation in COVID-19 patients and the pathogenesis of the hypercoagulable state including the role of antiphospholipid antibody. A regression analysis exploring predictive factors to the identification of PE will aid the pre-test probability, distinguishing those needing CTPA.

## Figures and Tables

**Table 1:** Laboratory features of cases 1–4 of COVID-19 patients presenting with an acute pulmonary embolus.

	Case 1	Case 2	Case 3	Case 4	Reference range
White cell count (10^9^/L)	10.10	8.82	9.68	13.49	3.5–11
Lymphocyte count (10^9^/L)	0.61	1.12	1.34	1.14	1–4
Neutrophil count (10^9^/L)	9.03	6.97	7.51	13.49	2–7.5
Platelet count (10^9^/L)	166	203	238	353	150–400
Prothrombin time (s)	11.1	13	11.9	11.4	10–13
Fibrinogen (g/L)	5.4	6.4	6.6	6.1	1.5–4.5
D-dimer (ng/mL)	9892	>80,000	23,068	11,448	<400
C-reactive protein (mg/L)	152	114	293	114	<5
Ferritin (ug/L)	1745	2197	1029	871	10–350
Troponin (ng/L)	27	25	65	15	<4
Alanine aminotransferase (u/L)	25	124	42	75	<42
N terminal probrain natriuretic peptide (ng/L)	229	N/A	1129	76	<300
Lactate (mmol/L)	1.67	2.11	6.85	1.45	<2
Partial pressure of oxygen (kPA) [mmHg] on 15 litres of oxygen	7.9 [60]	8.44 [63.3]	7.27 [54.5]	N/A	
Partial pressure of carbon dioxide (kPA) [mmHg] on 15 litres of oxygen	4.79 [35.9]	4.19 [31.425]	3.61 [27.1]	N/A	

*COVID-19*, coronavirus disease 2019; *L*, litre; *g*, gram; *ng*, nanogram; *mg*, milligram; *ug*, microgram; *mmol*, millimole; *kPa*, kilopascal; *mmHG*, millimetres mercury.

**Table 2:** Age, day of illness, suspicious clinical features, electrocardiogram findings, D-dimer value, ferritin value, and evidence of right heart strain in cases 1–4 and 2 cases in the literature.

	Case 1	Case 2	Case 3	Case 4	Danzi et al^2^	Casey et al^3^
Age	72	62	78	63	75	42
Day of illness	7	21	4	14	10	12
Suspicious clinical features	Hypotension Tachycardia	Tachycardia Delayed onset	Haemoptysis Tachycardia	Haemoptysis Pleuritic chest pain Tachycardia	Echocardiogram evidence of right heart strain	Chest pain Haemoptysis
ECG	Atrial fibrillation with rapid ventricular response	Sinus tachycardia	Atrial fibrillation with rapid ventricular response	Right bundle branch block	Normal sinus rhythm	S1Q3T3 pattern + right-axis deviation
D-dimer (<400 ng/L)	9,892	>80,000	23,068	11,448	21,000	4,800
Ferritin (10–350 ug/L)	1,745	2,197	1,029	871	N/A	N/A
Evidence of right heart strain	Nil	Nil	CTPA + echocardiogram	ECG + CTPA	Echocardiogram	ECG

*ECG*, electrocardiogram; *ng*, nanogram; *L*, litre; ug, microgram; *Nil*, none; *S1Q3T3*, deep S wave in lead I, a Q wave and inverted T wave in lead III; *CTPA*, computed tomography pulmonary angiogram.
